# Study of Complexity of Numerical Models of a Strengthened Timber Beam

**DOI:** 10.3390/ma16093466

**Published:** 2023-04-29

**Authors:** Michał Szczecina

**Affiliations:** Faculty of Civil Engineering and Architecture, Kielce University of Technology, Al. Tysiąclecia Państwa Polskiego 7, 25-314 Kielce, Poland; michalsz@tu.kielce.pl

**Keywords:** finite element method (FEM), wood–CFRP beam, Abaqus, numerical modeling

## Abstract

Laboratory research of wood–CFRP (carbon fiber reinforced polymer) structural elements, especially beams, is a scientific issue undertaken by many scientists. Research is often complemented with numerical analysis with the use of complex finite element method (FEM) models. Modern FEM software offers models that can reproduce such properties and phenomena as orthotropy and plasticity of wood and CFRP, delamination and mechanical behavior of adhesive layers, and damage of a strengthened element. The author of the paper reproduces numerical laboratory research of a four-point bending test of a glulam beam strengthened with CFRP tape. The main goal of the numerical research is an analysis of how the complexity of the FEM model influences the results of calculations, especially stress, deflection, and bearing capacity of the glulam beam. In some cases, a simpler model can be satisfactory, especially for a structural engineer, who takes into account serviceability limit states (permissible deflection of a structural member) and assumes that stress should not exceed the yield stress of timber.

## 1. Introduction

Structural elements made of wood and strengthened with various materials are a frequent subject of laboratory tests and numerical analysis. Research concerns different types of timber, for instance, veneer lumber [1], spruce timber [2], historic timber [3], glulam [4,5,6], as well as timber with knots and local deviation of grain [7]. Structural elements (mainly beams) are strengthened in various manners, e.g.,:

- With CFRP plates with different cross-sectional layouts [3,5,8];
- With CFRP cords [4];
- With screwed steel plates [9];
- As timber–glass composites (TGC) [10];
- With pultruded GFRP reinforcement [11];
- With basalt fibers [12,13];
- With jute fibers [14].

Laboratory tests are often accompanied by numerical simulations. Kawecki [15] presented a summary of guidelines for FEM modeling of CFRP–wood beams in the Abaqus environment. The guidelines take into consideration a few issues important in the numerical modeling of strengthened timber structures, namely:

- Constitutive law for wood, CFRP tapes, and adhesive,
- Cohesive behavior and traction separation of an adhesive layer,
- Material orientation,
- Proper recreation of damage and delamination,
- Convergence of solution of non-linear FEM problem.

Recently FEM calculation of timber structures using advanced numerical models has been a current and important topic of scientific interest. A few important papers on this topic are listed in this paragraph in chronological order. Mirianon et al. [16] presented a method to model wood in Abaqus code, which takes into consideration moisture flow and diffusion. Kim and Harries [17] discussed a parametric study of five timber species using orthotropic constitutive characteristics, analyzing stress concentration and failure modes. Raftery and Harte [18] developed a numerical model that incorporated non-linear material behavior and non-linear geometry as well. Glisović et al. [5] defined a numerical model using anisotropic plasticity theory and maximum stress criterion for failure. However, they defined an adhesive bond between timber and CFRP plates as a perfect connection. In the previously mentioned work of Kawecki [15], the same bond was defined with the use of the so-called “cohesive behavior” in the ABAQUS code. As the author claims, the definition is intended to better reproduce the delamination process. There is also an effort put into the numerical simulation of the hygro-mechanical creep behavior of strengthened timber elements, as shown in [19]. The authors used DFLUX and UMAT subroutines to describe the relative humidity and material behavior. Kawecki and Podgórski [20,21] developed numerical models which recreated fractures in softwood bent elements (using linear elastic fracture mechanics, continuum damage mechanics, and Hill’s function) and examined the effect of glue cohesive stiffness on the elastic response of timber beams. Eslami et al. [22] proposed a non-linear anisotropic material model for failure and its implementation in the UMAT subroutine in the ABAQUS environment.

The vast majority of scientific analyzes of the statics of timber elements assume an elastic–plastic material model. A simple version of the elastic–plastic model assumes yielding in the compressive zone [5], which is consistent with design codes [23]. One can also define plastic behavior separately in tension and compression, which was demonstrated in [15,20]. The other approach is a separate definition of the elastic–plastic model in three directions: parallel to grain, radial, and tangential direction [18]. It is also possible to incorporate damage formulations with damage criteria in tension and compression, as shown in [22]. Elastic models in scientific research are rarely used; an example of the use of the elastic model can be found in work by Braun et al. [24]. However, even in that case, the authors reported some restrictions when using the elastic model. On the other hand, design codes [23] recommend the use of the elastic model or the elastic–plastic model with a definition of yielding in the compressive zone only. Structural engineers often use the simplest (so elastic) model and may not be aware of the disadvantages of this model. There is a need to perform analyses that compare the behavior of the elastic and elastic–plastic models and determine how complex should be a FEM model to reflect a real behavior of a timber structural element. A novelty of the presented work is based on the comparative study of assumed numerical models.

Numerical modeling of timber structures encounters a few relevant issues, which are highlighted in this paragraph. In general, the more accurate the model is, the more complicated phenomena should be implemented in the FEM software. The very first problem is a choice of a material model for timber. Brol et al. [25] stated that the assumption of timber as homogenous material leads to the neglection of irregularities in the material. Because of that, an assumption of orthotropic material is widely used. As an anisotropic yield criterion, Hill’s criterion is most often assumed, as it was developed from the Huber–Mises–Hencky (HMH) criteria and allows consideration of different material behavior in three orthogonal directions [26], even with the distinction between compressive and tensile behavior of the material. The other issue concerns the proportion of dimensions of timber, adhesive, and CFRP plates. Relatively small thickness of adhesive and CFRP layers may cause some numerical problems. An exemplary solution to the problem was proposed by Kawecki [27], who introduced a unit constitutive thickness for the cohesive element and surfaces. The next problem refers to a definition of connections between timber, adhesive, and CFRP plates. There are two approaches; one of them is a simple perfect bond between all items [3,5]. Of course, this means the neglection of phenomena in adhesive layers. The other approach, presented, e.g., by Kawecki [27], assumes an introduction of the traction–separation law. Finally, some problems with convergence can occur during non-linear FEM analysis. A solution to the problem was proposed in [27] by inputting a viscosity parameter, which guarantees the stability of the model.

To sum up, current trends in research of strengthened timber elements demand a comparison of laboratory and numerical research. Numerical models become more complicated in the sense that they can reproduce more and more complex phenomena. The author of the paper would like to present the results of the FEM calculations and compare them to the experimental results presented in [5]. The main goal of the paper is a review of results depending on the complexity of the FEM model; therefore, it can be considered a numerical study (a FEM calculation report). The complexity manifests itself with the following issues: choice of a material model (elastic or elastic–plastic) and definition of a cross-section of the glulam beam (whole section or division into laminations). Results obtained in an elastic model (taking into account a serviceability limit state, and namely permissible deflection in the middle span of the beam) can have importance to structural engineers, while more complex models can be of interest to both engineers and researchers.

## 2. Materials and Methods

### 2.1. Materials

**Figure 1:**
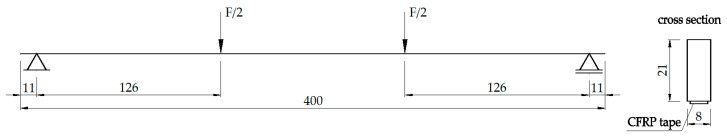
Static scheme of the specimen and its cross-section (dimensions in [cm]) [5].

**Figure 2:**
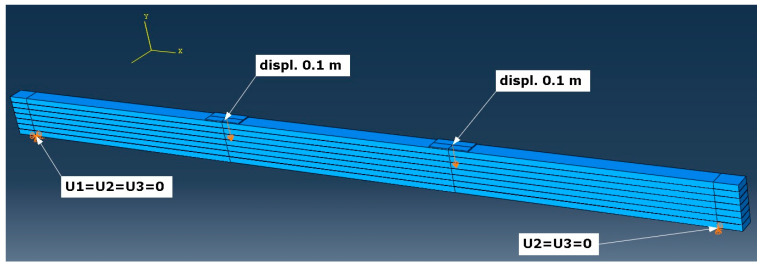
Boundary conditions and load of the specimen.

**Figure 3:**
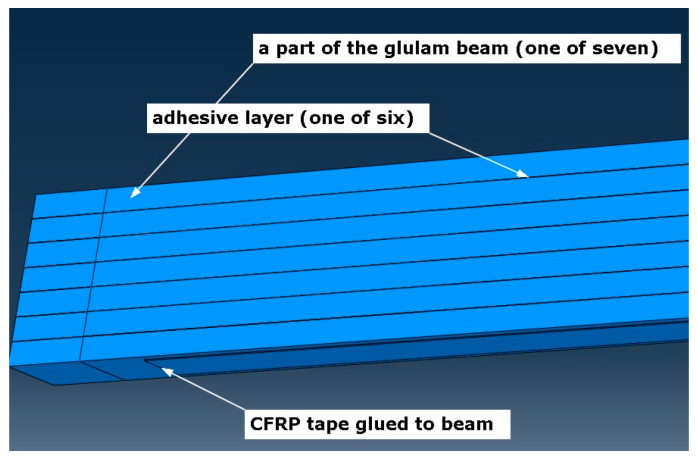
Parts creating the whole specimen.

**Figure 4:**
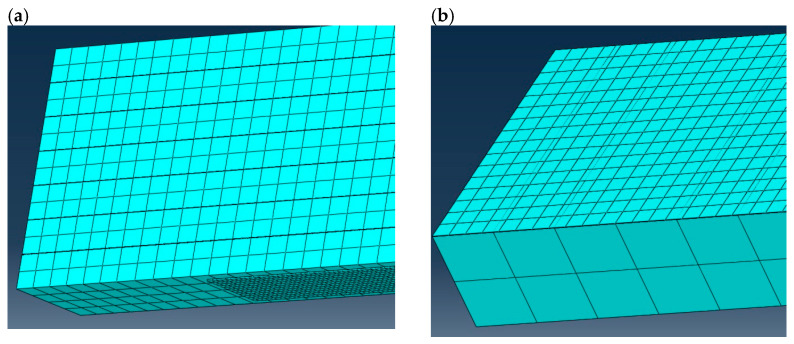
Meshing of the specimen (**a**) a zoomed support zone, (**b**) a part of the glulam beam with meshed adhesive layer.

Numerical simulations were carried out on a glulam beam under four-point bending, strengthened with CFRP tapes. Geometry (see Figure 1), mechanical properties, and boundary conditions were assumed according to [5]. Boundary conditions and load of the specimen (displacement control was applied) are shown in Figure 2. Maximal displacement of the indicated nodes was assumed as 0.1 m. Translational degrees of freedom U1, U2, and U3 affiliated with the x-, y-, and z-axis of the global coordinate system, respectively (Figure 2). The assembled specimen is presented in Figure 3. The whole timber section consists of seven parts (laminations) which are connected by six adhesive layers. A CFRP tape is glued with another adhesive layer to the bottom of the glulam beam. Meshing of the specimen is shown in Figure 4, wherein Figure 4a) presents a zoom of a support zone (one can see a finer mesh defined for the CFRP tape and a coarser mesh applied for the glulam beam) and Figure 4b) shows a zoom of one part of the glulam beam with an adhesive layer.

Material properties of timber and CFRP are listed in Table 1 and Table 2 according to [5]. Timber and CFRP were assumed as orthotropic, while adhesive layer is isotropic and linear–elastic with modulus of elasticity equal to 11,200 MPa and Poisson’s ratio 0.35, according to a manufacturer of the adhesive [5]. A few mechanical properties of materials (in the case of timber—parallel to grain), especially those valid for definition of constitutive relationships, were presented in Table 3 [5].

### 2.2. Methods

Calculations were performed with FEM using Abaqus [28] environment. Four variants of a model with different assumptions were analyzed. The variants are listed in Table 4. The glulam beam taken into consideration consists of seven parts glued with adhesive layers, and the first question is if one can simply define a whole timber cross-section without dividing it into seven glued laminations. One of goals of the research is to check out if the cross-section definition has any significant influence on obtained results.

The more important problem concerns a material model definition of timber. There is no doubt it should be an orthotropic model [15], but the open issue is the use of plastic behavior in timber. From a structural engineer’s point of view, codes recommend employing a linear elastic model of timber, as, for example, stated in Eurocode [23]. The code mentions only compressive behavior of timber; more precisely, it allows using a non-elastic model in compression but without any specific constitutive equation. It is very likely that structural engineers use linear–elastic models without any consideration of plastic behavior of timber. On the other hand, a comparison of results obtained using FEM for both models (linear elastic and plastic) combined with SLS (serviceability limit state), especially with a verification of a permissible deflection in the midspan of the glulam beam, can be an answer to an engineering issue if the use of the plastic model is necessary, whereas, when comparing laboratory tests with numerical simulations, the plastic model (often combined with cohesive behavior of an adhesive layer to reproduce delamination—see [15]) is a more correct option.

Timber and CFRP tapes as orthotropic materials were defined using “Elastic–engineering constants” option in Abaqus. This option allows inputting all nine material constants listed in Table 2 and Table 3. To reproduce an anisotropic plastic flow of timber (when reaching a yield point), Hill’s function is applied—see Equation (1):

(1) $$f\left(\sigma \right)=\sqrt{F{\left(\sigma_{22}-\sigma_{33}\right)}^{2}+G{\left(\sigma_{33}-\sigma_{11}\right)}^{2}+H{\left(\sigma_{11}-\sigma_{22}\right)}^{2}+2N\tau_{12}^{2}+2M\tau_{13}^{2}+2L\tau_{23}^{2}}$$

where $\sigma_{ij}$ and $\tau_{ij}$ denote components of a stress tensor σ and six constants appearing in the equation can be expressed in the form given by Equations (2) and (3):

(2) $$F=\frac{1}{2}\left(\frac{1}{R_{22}^{2}}+\frac{1}{R_{33}^{2}}-\frac{1}{R_{11}^{2}}\right),\;G=\frac{1}{2}\left(\frac{1}{R_{33}^{2}}+\frac{1}{R_{11}^{2}}-\frac{1}{R_{22}^{2}}\right),\;H=\frac{1}{2}\left(\frac{1}{R_{11}^{2}}+\frac{1}{R_{22}^{2}}-\frac{1}{R_{33}^{2}}\right),$$

(3) $$N=\frac{3}{2R_{12}^{2}},\;M=\frac{3}{2R_{13}^{2}},\;L=\frac{3}{2R_{23}^{2}},$$

where constants were calculated using formulas given by Abaqus guide [28] and yield points assumed in [5]. The formulas and values of the six constants are listed below:

(4) $$R_{11}=\frac{{\bar{\sigma }}_{11}}{\sigma^{0}}=\frac{36.30MPa}{36.30MPa}=1,$$

(5) $$R_{22}=\frac{{\bar{\sigma }}_{22}}{\sigma^{0}}=\frac{5.00MPa}{36.30MPa}=0.138,$$

(6) $$R_{33}=\frac{{\bar{\sigma }}_{33}}{\sigma^{0}}=\frac{5.00MPa}{36.30MPa}=0.138,$$

(7) $$R_{12}=\frac{{\bar{\sigma }}_{12}}{\tau^{0}}=\frac{6.10MPa}{21.96MPa}=0.291,$$

(8) $$R_{13}=\frac{{\bar{\sigma }}_{13}}{\tau^{0}}=\frac{6.10MPa}{21.96MPa}=0.291,$$

(9) $$R_{23}=\frac{{\bar{\sigma }}_{23}}{\tau^{0}}=\frac{3.00MPa}{21.96MPa}=0.143,$$

where $\tau^{0}=\frac{\sigma^{0}}{\sqrt{3}}$. All the *R_ij_* constants are listed in Table 5 and in this order they should be input while defining Hill’s function in Abaqus environment.

**Figure 5:**
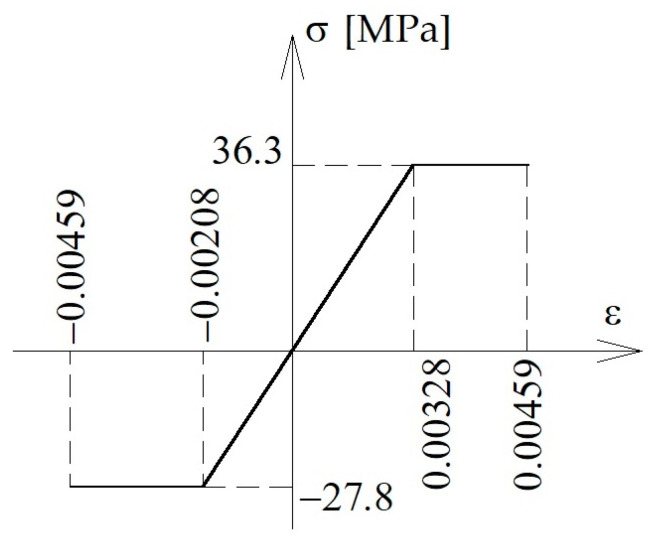
Stress–strain relationship assumed in numerical simulations.

Plastic properties of timber were defined using classical plasticity option in Abaqus code. The ideal elastic–plastic model was assumed, as shown in Figure 5, with maximal tensile stress equal to 4.59‰ (see series B in [5]). Yield strains at tension and compression (at the beginning of the plastic process) were calculated as follows (Equations (10) and (11)):

(10) $$\varepsilon_{y,t}=\frac{27.8MPa}{11080MPa}=0.00251,$$

(11) $$\varepsilon_{y,c}=\frac{36.3MPa}{11080MPa}=0.00328.$$

All the parts were modeled with the C3D8R finite elements (8-node linear brick with reduced integration and enhanced hourglass control). All interaction properties between timber, adhesive layers, and CFRP tapes were applied as so-called “tie” in Abaqus code, so a full bond between all parts was defined.

## 3. Results

To compare laboratory tests [5] with numerical analysis, the author of the paper decided to present the following results (all maps at the last step of FEM calculations):

- Stress distribution in timber and CFRP tape;
- Force–deflection relationship for a point in the mid-span of the beam;
- Shear stress in adhesive layers.

**Figure 6:**
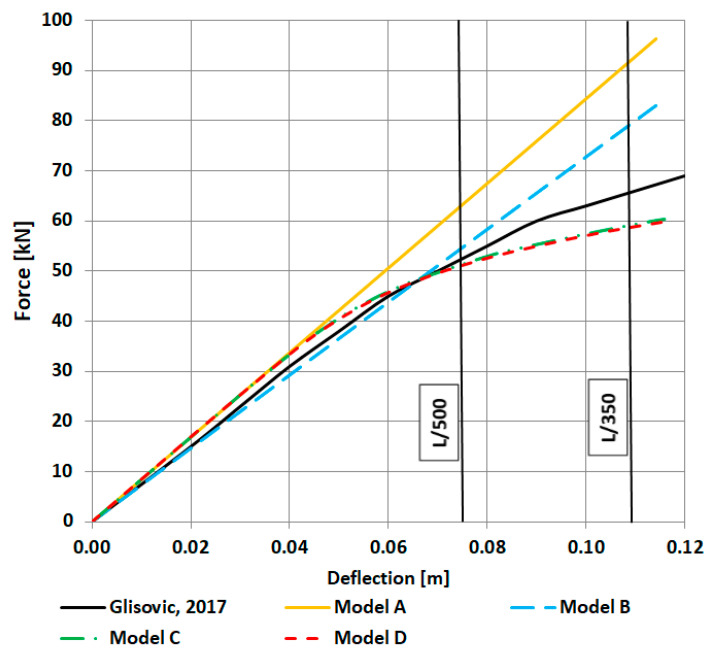
Force–deflection relationship obtained in numerical simulations and compared with laboratory tests [5] (results for models C and D almost coincide).

The force–deflection relationship is presented in Figure 6. Deflection was measured in the mid-span and at the bottom of the beam. Two typical permissible deflections (L/500 for lintels and L/350 for main beams or joists, where L denotes the span of the beam; in this paper, L = 3.78 m) are marked with vertical lines. We can see that model A responds with a higher stiffness even for small values of deflection. The observation means that a linear elastic model of a glulam beam with no division of the cross section into laminations is not a proper option to model this structural element. In the case of model B, the response is better and similar to models C and D, but only limited to small deflection (L/500). Finally, models C and D show a very similar response, and both are comparable with laboratory tests of Glisović et al. [5]. In the following part of the paper, results in the form of maps and graphs obtained in models A–D are presented. Moreover, for the sake of the visibility of the presented maps, only half of the model is shown (results are symmetric).

**Figure 7:**
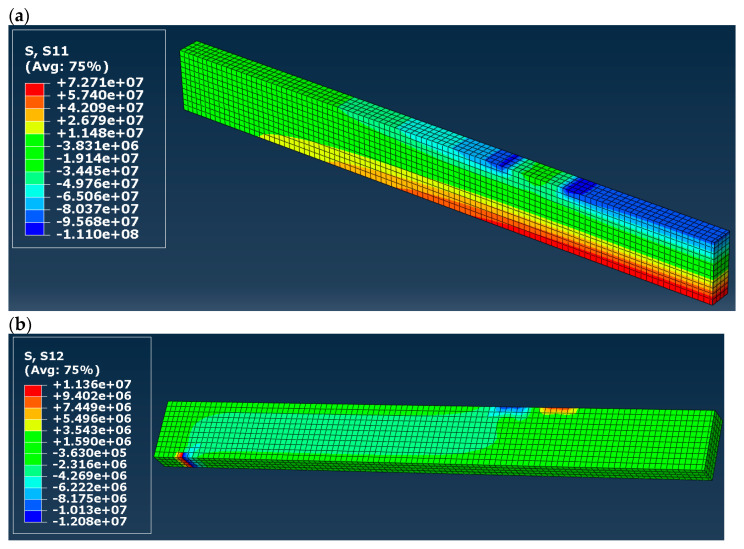
Stress distribution in timber in model A (**a**) normal (S11) and (**b**) shear (S12), results in [Pa].

**Figure 8:**
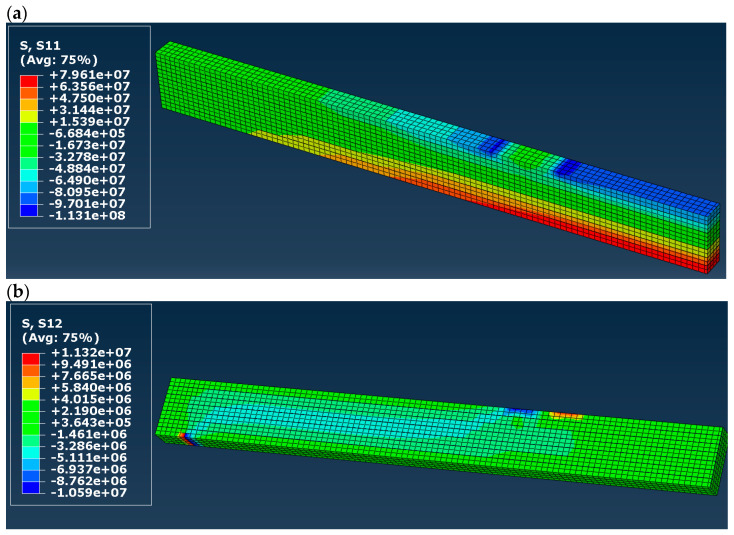
Stress distribution in timber in model B (**a**) normal (S11) and (**b**) shear (S12), results in [Pa].

**Figure 9:**
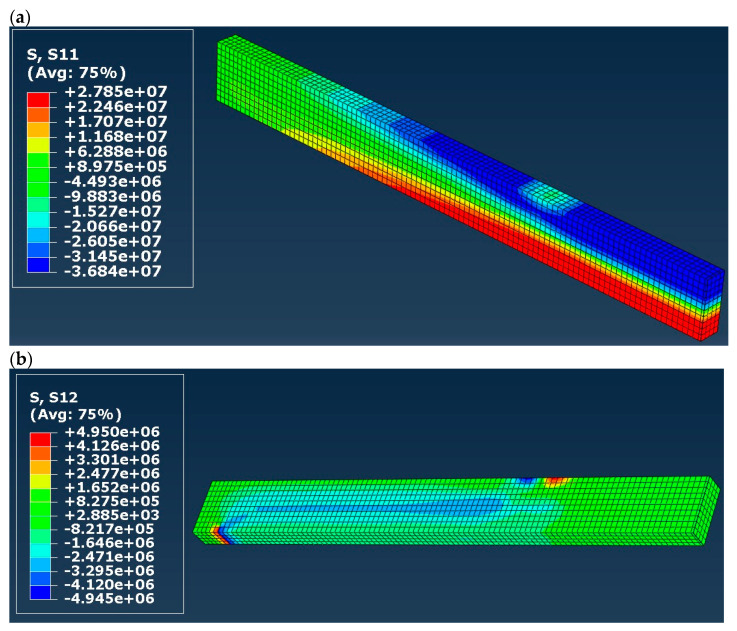
Stress distribution in timber in model C (**a**) normal (S11) and (**b**) shear (S12), results in [Pa].

**Figure 10:**
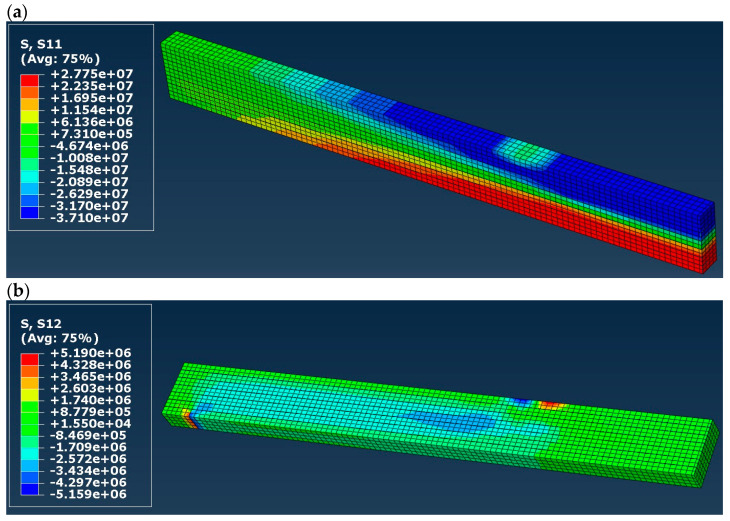
Stress distribution in timber in model D (**a**) normal (S11) and (**b**) shear (S12), results in [Pa].

Normal (S11 in the presented maps) and shear (S12) stress distribution in timber in models A to D is shown in Figure 7, Figure 8, Figure 9 and Figure 10. Results are expressed in [Pa] and notation, e.g., 10^6^ denotes 10 to the power of 6. In the case of models A and B (elastic), both normal and shear stress are much higher than in the case of models C and D. In model B, the normal stress reaches almost 80 MPa in the tension zone and 113 MPa in the compression zone, which are values widely higher when comparing them to the compressive and tensile strength of timber (36.3 MPa and 27.8 MPa respectively). That means that models A and B do not reflect reality. In the case of models C and D (elastic–plastic) we can see that in tension as well as in a compression zone, normal stress reached a value of yield stress, and the yielding of timber occurred in large regions of the glulam beam (red and blue color in the maps). In practice, this means damage to the structural element, which also occurred in laboratory tests by Glisović et al. [5]. Moreover, general patterns of normal and shear stress are comparable with those presented in [5] (see Figure 10 in [5]).

**Figure 11:**
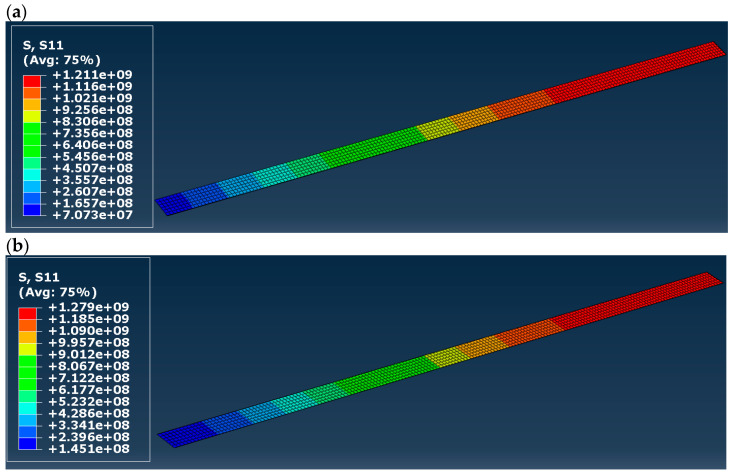

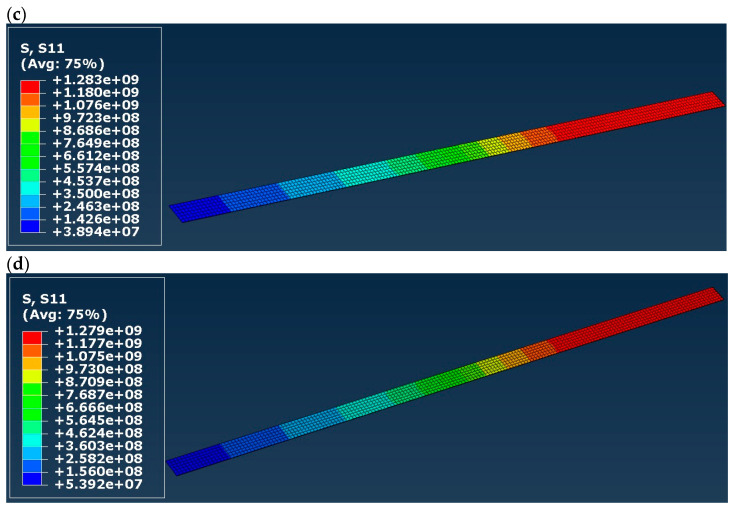
Normal (S11) stress distribution in the CFRP tape: (**a**) in model A, (**b**) model B, (**c**) in model C, (**d**) in model D; results in [Pa].

Normal stress distribution (S11) in the CFRP tape is presented in Figure 11, separately for all the models. The distribution in all cases is practically identical, with the highest value in the range of 1.21 to 1.28 GPa, which is far lower than the tensile strength presented in Table 3. Glisović et al. [5] obtained a very similar distribution but a smaller value of the maximal normal stress (0.86 GPa—see Figure 12a) in [5]). It does not change the fact that the CFRP did not damage according to FEM calculations, regardless of the applied material model of timber (elastic or elastic–plastic).

**Figure 12:**
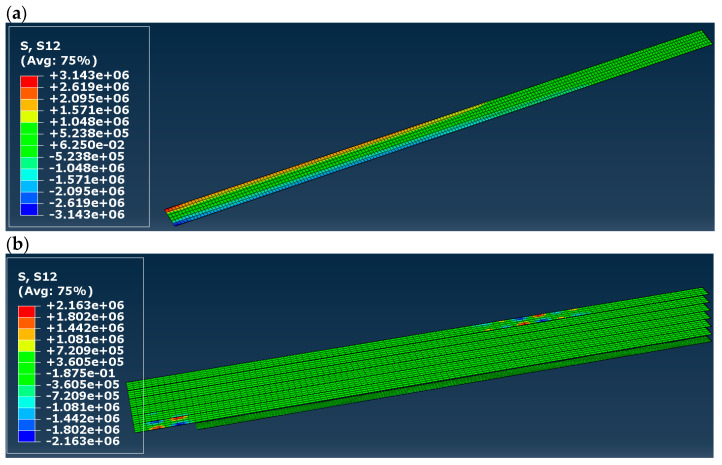

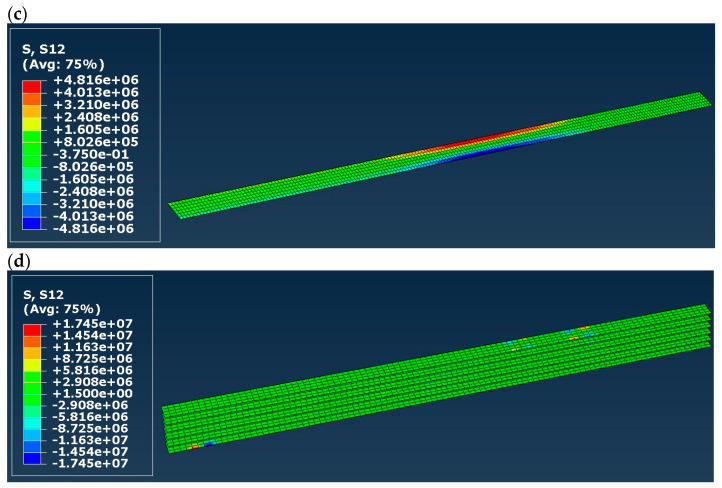
Shear (S12) stress distribution in the adhesive layer(s): (**a**) in model A, (**b**) model B, (**c**) model C, (**d**) model D; results in [Pa].

Shear stress (S12) in adhesive layer(s) is presented in Figure 12. In the case of models B and D, the maps in Figure 12b,d present all adhesive layers in the model, while in the case of model C, the only adhesive layer is the one between the timber beam and the CFRP tape. In models A to C, the highest value of shear stress differs from 2.16 to 4.82 MPa, and it is significantly lower the shear strength of the adhesive layer (18 MPa). Moreover, the value obtained in model C is comparable with the one presented in [5] (4.30 MPa). In the case of model D, the highest value of shear stress is 17.45 MPa, and it occurs in the support zones. It is still lower than the shear strength of the adhesive layer, so no delamination occurs, according to the FEM models.

**Figure 13:**
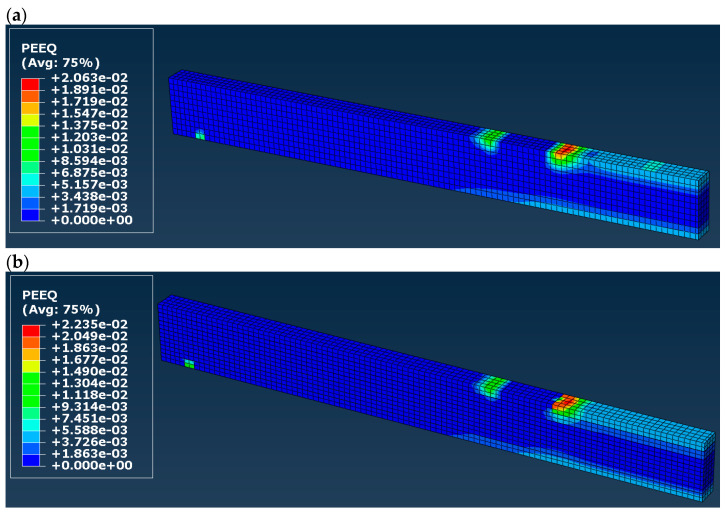
Equivalent plastic strains (PEEQ) obtained in the (**a**) model C, (**b**) model D.

In order to make sure that there are plastic deformations in models C and D, the maps of an equivalent plastic strain (PEEQ in the Abacus code) were plotted and presented in Figure 13. We can see large values of the equivalent plastic strains in the boundary condition zones (i.e., supports and external forces). Lower but still plastic strains are marked with a light-blue color in the mid-span of the beam (both in the tension and compression zones). This means that there are actually plastic deformations in the model (which will not disappear after removing the load of the beam), and therefore, the elastic–plastic orthotropic model seems to be more accurate in reproducing the beam using FEM.

**Figure 14:**
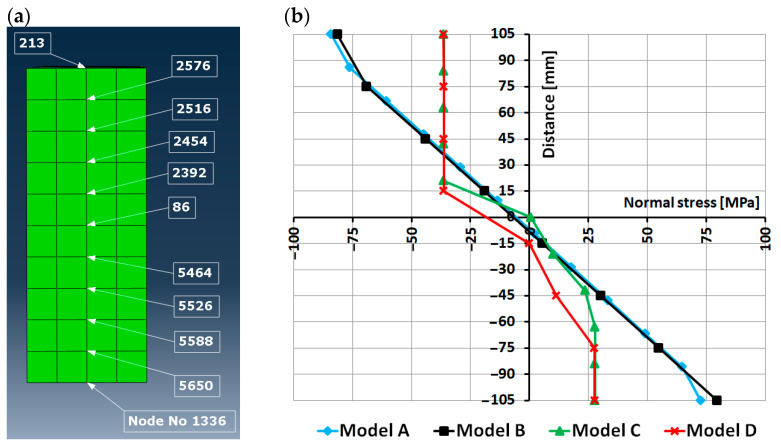
Cross-section in the mid-span of the beam: (**a**) division of the cross-section by example of model C; (**b**) Normal stress (S11) distribution in the cross-section.

Finally, a distribution of the normal stress (S11) in the cross-section of the beam was analyzed for all the models. The cross-section was chosen exactly in the mid-span of the beam, where the highest values of the bending moment and deflection are expected. A sample scheme of a few points chosen to plot the normal stress graph is presented in Figure 14a); the scheme is presented for model C. A division of the cross-section height into calculation points differed depending on the model (please note that for models B and D, the cross-section is divided into laminates, so the numbering and amount of calculations point can be different for the different models). The node is numbered as 1336 in Figure 14a) is the one in which the deflection of the beam was controlled in the numerical analysis. The graph of the normal stress versus the distance from the center of symmetry of the rectangular timber section is presented in Figure 14b). Please note that negative coordinates of the distance mean that a calculation point lies below the center of symmetry. A negative value of normal stress denotes compressive stress. As we can see, the stress distribution in the case of models A and B is purely elastic and almost identical. As mentioned above, the values of the normal stress are much higher than the strength of timber applied in the model. The stress distribution for models C and D shows a large yielding of timber, especially in the compression zone. Both graphs look similar, and the only difference is the distribution of the stress apart from the yielding region.

## 4. Discussion

Results obtained using FEM simulations leave no doubt that a linear elastic model is not a proper one to analyze a glulam beam strengthened with CFRP tapes. As reported in [5], such beams (in laboratory tests) show partial yielding with a non-linear segment of a force–deflection relationship, and laminations remain intact. The use of the linear elastic model leads to an overestimation of the stiffness of the glulam beam (see Figure 6) and to an overestimation of normal stress, too (Figure 14). The other important issue is a proper definition of a cross-section of the beam. When the cross-section is not divided into laminations (glued with adhesive layers), the linear–elastic response is even stiffer. Dividing into the laminations makes the force–deflection relationship more realistic, but only in the case of relatively small deflections (no larger than L/500, where L denotes a span of the structural element). On the other hand, the use of a plastic model (with different yielding conditions in tension and compression) allows the reproduction of the laboratory test well—as shown in Figure 6, the response is even more conservative than the one obtained in laboratory tests [5]. In the case of the plastic model, there is no significant difference in the definition of the cross-section. Of course, if one wants to reproduce shear stress in the adhesive layers, division into the laminations is obligatory. To sum up, modern FEM codes allow us to take into account more complex material models of glulam elements, CFRP tapes, and adhesives. The author of the paper believes that design codes and handbooks of the structural design of timber should also respond to the need for supplementation of material models. Plastic models should take into account not only the compressive but also the tensile behavior of timber.

A brief comparison of all four models is presented in Table 6. Models C and D seem to be the most useful for FEM calculations of a timber beam strengthened with CFRP tapes. There is no significant difference in results if one divides the cross-section of the beam into laminations or not. A choice of the proper material model is clear—it should be the elastic–plastic model. A division into the tensile and compressive plastic behavior of timber is recommended. A very simple FEM model with the assumption of the elastic behavior of timber leads to an overestimation of normal stress and deflection. Structural engineers should be aware of these issues and should not try to make their model too simple (as Albert Einstein said: “Make everything as simple as possible, but not simpler”).

The results presented in this paper can also be compared (in a qualitative sense) with the work of Nowak et al. [3]. The authors also compared laboratory tests and numerical simulations and also used an elastic and an elastic–plastic model. Equilibrium paths obtained in their work using FEM software (see Figures 10–14 in [3]) are similar to the graphs presented in Figure 6 in this paper. It is clear that in the case of the elastic model, a typical plateau in the graph is not reproduced. Only the use of the plastic model allows a better match to experimental results. A more interesting observation concerns a distribution of normal stress in a cross-section of the beam. The above-mentioned authors (see Figure 11 and Figures 15–17 in [3]) obtained a linear distribution of the normal stress and, moreover, the graphs for the elastic and the elastic–plastic model coincide each other. In this paper, in Figure 14, we can also see the linear stress distribution, but only in the case of the elastic model, whereas the elastic–plastic model recreates a presence of relatively large yielding zones, both in tension and compression. In the author’s opinion, the elastic–plastic model used in this work reproduces the normal stress distribution in a better way. Generally speaking, the numerical models C and D adopted by the author allow us to reproduce the laboratory test quite well and ensure a realistic distribution of the normal stress.

Finally, there are some issues that should be pondered in future work. The first issue is a definition of a constraint between all consecutive parts forming a glulam element. In this paper, the author proposed a simple full-bond connection called “tie” in the Abaqus code. As presented in the paper, this simplified definition did not cause any significant divergence in comparison with laboratory tests (in models C and D, where the plastic model was applied). On the other hand, in that case, the delamination process can be reproduced only with an analysis of shear stress in the adhesive layers. Kawecki [15] proposed a definition of the so-called cohesive finite elements in the Abaqus code. The author of this paper sees that option as the next step of his on-going work with FEM simulations of glulam beams. However, it should be taken into account that this kind of non-linear effects in a FEM model (contact and decohesion) can lead to some numerical problems (even if a FEM solver assumes very small initial steps to make the contact conditions “visible” for it in the model). A guideline on how to cope with such issues was presented in [15]. Moreover, the definition of contact (as cohesive elements) demands choosing a law that defines damage initiation. Some material constants (e.g., energy for energy-based damage criteria) should be calibrated and input into the model. The other issue is the incorporation of damage criteria, separately for tension and compression. The author of the paper intends to ponder these two issues (contact and damage criteria) in future work. As presented in this paper, the simplified model can be sufficient to design a glulam beam.

## 5. Conclusions

Based on the FEM calculation results and the author’s hitherto experience, the following conclusions can be drawn:

- An orthotropic linear elastic model seems not to be a proper model to reproduce the mechanical response of a glulam structural element; structural engineers should be aware of this fact when designing a structural element made of timber strengthened with CFRP tapes (the model can be valid only for relatively small values of mid-span deflection);
- Exceptionally, an orthotropic linear elastic model in the case of a cross-section divided into laminates (model B in the paper) can reproduce reality quite well, but it is limited to small values of deflection of a glulam beam;
- An orthotropic elastic–plastic model behaves well in comparison to a laboratory test; generally speaking, a structural engineer, when designing a timber glulam beam strengthened with CFRP tapes, should seriously consider the use of the plastic model;
- A division of a cross-section into laminates does not significantly affect the results of the FEM calculations.

The study presented in this paper assumes a simplified numerical model of a strengthened timber element. Further FEM calculations should take into account a different interaction between adhesive, timber, and tapes, for example, cohesive finite elements. This modification can reproduce delamination better than in the models presented in this paper. The other issue for future work is a definition of damage criteria, both in tension and compression.

## Figures and Tables

**Table 1:** Orthotropic material properties of timber.

Modulus of Elasticity [MPa]			Shear Modulus [MPa]			Poisson’s Ratio [-]		
E_1_	E_2_	E_3_	G_12_	G_13_	G_23_	ν_12_	ν_13_	ν_23_
11,080	886	554	791	744	79	0.37	0.42	0.47

**Table 2:** Orthotropic material properties of CFRP tape.

Modulus of Elasticity [MPa]			Shear Modulus [MPa]			Poisson’s Ratio [-]		
E_1_	E_2_	E_3_	G_12_	G_13_	G_23_	ν_12_	ν_13_	ν_23_
165,543	10,000	10,000	5000	5000	1000	0.30	0.30	0.03

**Table 3:** Mechanical properties of materials.

Property	Timber	CFRP	Adhesive Layer
Compressive strength [MPa]	36.3	-	70.0
Tensile strength [MPa]	27.8	2846.0	24.0
Bending strength [MPa]	42.5	-	-
Shear strength [MPa]	-	-	18.0

**Table 4:** Different model assumptions.

Model Name	Cross-Section	Timber Definition
A	whole section, no division	orthotropic, linear elastic
B	divided into 7 parts, glued with adhesive	orthotropic, linear elastic
C	whole section, no division	orthotropic, plastic
D	divided into 7 parts, glued with adhesive	orthotropic, plastic

**Table 5:** Constants *R_ij_* assumed for FEM simulation.

R_11_	R_22_	R_33_	R_12_	R_13_	R_23_
1.000	0.138	0.138	0.291	0.291	0.143

**Table 6:** Comparison of all investigated models.

Criterion	Model A	Model B	Model C	Model D
Force vs deflection curve	Overestimated, no plateau		Similar to laboratory tests, with plateau	
Normal stress in timber	Very overestimated		Restricted to the yield stress value, maps similar to [5]	
Shear stress in timber	Overestimated, but maps similar to [5] in a qualitative sense		Maps and values similar to [5]	
Normal stress in CFRP tapes	Practically identical in all models, below the tensile strength and clearly higher than in the laboratory tests			
Shear stress in adhesive layer(s)	Clearly below the shear strength, no delamination		Similar to the laboratory tests, clearly below the shear strength, no delamination	Clearly higher than in the tests, but still below the shear strength, no delamination
Plastic strains	(not applicable)		Very similar in both models, concentration in the support and loading zones	

## Data Availability

Data sharing is not applicable to this article.
